# Diagnostic performance of prospective same-day 18F-FDG PET/MRI and 18F-FDG PET/CT in the staging and response assessment of lymphoma

**DOI:** 10.1186/s40644-023-00520-7

**Published:** 2023-01-24

**Authors:** Vijay Mistry, Justin R. Scott, Tzu-Yang Wang, Peter Mollee, Kenneth A. Miles, W. Phillip Law, Greg Hapgood

**Affiliations:** 1 Department of Medical Imaging, Princess Alexandra Hospital, Brisbane, Australia; 2 QCIF Bioinformatics, Institute for Molecular Bioscience, The University of Queensland, Brisbane, Australia; 3 Department of Haematology, Princess Alexandra Hospital, Brisbane, Australia; 4 Translational Research Institute, Princess Alexandra Hospital, Brisbane, Australia; 5 Institute of Nuclear Medicine, University College London, University College Hospital, London, UK; 6 School of Medicine, University of Queensland, Brisbane, Australia

**Keywords:** Hodgkin lymphoma, Non-Hodgkin lymphoma, PET/MRI, PET/CT, Lymphoma, 18F-FDG

## Abstract

**Background:**

Accurate staging and response assessment are essential for prognosis and to guide treatment in patients with lymphoma. The aim of this study was to compare the diagnostic performance of FDG PET/MRI versus FDG PET/CT in adult patients with newly diagnosed Hodgkin and Non- Hodgkin lymphoma.

**Methods:**

In this single centre study, 50 patients were prospectively recruited. FDG PET/MRI was performed after staging FDG PET/CT using a single injection of 18F-FDG. Patients were invited to complete same-day FDG PET/MRI with FDG PET/CT at interim and end of treatment response assessments. Performance was assessed using PET/CT as the reference standard for disease site identification, staging, response assessment with Deauville score and concordance in metabolic activity.

**Results:**

Staging assessment showed perfect agreement (κ = 1.0, *P* = 0) between PET/MRI and PET/CT using Ann Arbor staging. There was excellent intermodality correlation with disease site identification at staging (κ = 0.976, *P* < 0.001) with FDG PET/MRI sensitivity of 96% (95% CI, 94–98%) and specificity of 100% (95% CI, 99–100%). There was good correlation of disease site identification at interim assessment (κ = 0.819, *P* < 0.001) and excellent correlation at end-of-treatment assessment (κ = 1.0, *P* < 0.001). Intermodality agreement for Deauville scores was good at interim assessment (κ = 0.808, *P* < 0.001) and excellent at end-of-treatment assessment (κ = 1.0, *P* = 0). There was good–excellent concordance in SUV max and mean between modalities across timepoints. Minimum calculated radiation patient effective dose saving was 54% between the two modalities per scan.

**Conclusion:**

With high concordance in disease site identification, staging and response assessment, PET/MR is a potentially viable alternative to PET/CT in lymphoma that minimises radiation exposure.

## Introduction

Accurate staging and response assessment are essential for prognosis and to guide treatment in patients with lymphoma. 18F-2-deoxy-2-fluoro-D-glucose positron emission tomography (FDG-PET)/CT has become the standard of care in the staging and response assessment for FDG-avid Hodgkin and non-Hodgkin lymphomas [1–5]. There is now considerable experience with the prognostic value of PET/CT in lymphoma with interim and end of treatment assessments characterised by a Deauville score [6–10]. This is a 5-point score where a quantitative and qualitative assessment of standardised uptake values (SUV) in residual lymphoma lesions are compared with the SUV in mediastinal blood pool and liver allowing a comparison of change in SUV from baseline [11].

Patients diagnosed with lymphoma typically require multiple imaging examinations over the course of diagnosis, treatment and post-remission surveillance. Considering the potential impact of radiation exposure and the potential for second cancer, imaging techniques which minimise radiation exposure, such as magnetic resonance (MRI) imaging, are of clinical interest [12, 13].

The introduction of hybrid PET/MRI imaging systems has allowed simultaneous acquisition of PET and MRI data, providing anatomical and functional information with exquisite soft tissue contrast. PET/MRI has been shown to detect sites of disease in patients with lymphoma with comparable sensitivity to PET/CT in adult [13–15] and paediatric [16, 17] populations. Multiple studies have suggested that PET/MRI is comparable to PET/CT for staging lymphoma [13, 14, 18–20]. A smaller number of studies have recently prospectively evaluated both staging and response assessment and reported comparable Deauville scores between PET/MRI and PET/CT in lymphoma patients in adults [15] and children [17].

Quantitative parameters can be used to measure the radioactivity within a lesion. SUV parameters have shown concordance between the two modalities in a broad range of malignancies [21] as well as lymphoma staging assessments [13, 14, 17, 20]. Limited data exists in lymphoma comparing metabolic activity (SUVmax/SUVmean) between FDG PET/MR and PET/CT in physiological and pathological uptake across treatment timepoints [15].

The aim of this prospective study was to compare the diagnostic performance of same day FDG PET/MRI versus FDG PET/CT in adult patients with newly diagnosed lymphoma. The hypothesis was that FDG PET/MRI would represent a practical alternative to FDG PET/CT in the identification of disease sites, staging and response assessment in lymphoma, with reduced exposure to radiation.

## Materials and methods

### Patient population

This prospective single-centre study was performed at the Princess Alexandra Hospital, a tertiary referral centre for a large health district. Patients were invited to participate if they had a new diagnosis of any subtype of lymphoma and were aged ≥ 18 years. Patients were excluded if they were pregnant or had a contraindication or inability to tolerate PET/MRI. Patients were recruited from all patients routinely referred to our institution for PET/CT. Patients underwent same day PET/MRI following PET/CT for staging purposes using the same 18F-FDG injection. All patients were invited to complete a PET/MRI with their PET/CT at interim and end of treatment PET/CT assessments. The protocol was approved by the local ethics committee and all patients provided informed written consent.

### Image acquisition

All patients fasted for at least six hours prior and serum glucose was confirmed to be less than 11 mmol/L prior to body weight adjusted intravenous administration of FDG (4 MBq/kg to a maximum of 360 MBq). All subjects received a single intravenous injection of FDG followed by an uptake phase of 60 min prior to PET/CT. 90 min after the injection of FDG radiotracer, subjects underwent PET/MRI following the initial PET/CT.

### 18F-FDG PET/CT Protocol

PET/CT was performed on a hybrid PET/CT scanner combining PET with a 128-slice multi-detector CT Siemens Biograph 128mCT (Siemens Healthcare, Germany). Low-radiation-dose CT for attenuation correction was performed from the skull base to mid thighs using the following parameters: 120 kV, Qref set to 80 mAs, 0.5 s rotation time, pitch 1.0, 32 × 1.2 mm detectors selected with 5-mm reconstruction, acquired with an extended field of view of 780 mm, matrix size 512 × 512 and pixel separation 0.98mmx 0.98 mm. Axial CT slices were reconstructed with standard filter kernel and lung algorithms. PET emission scan was obtained over the same anatomical area (skull vertex to mid thighs). PET acquisitions were assessed on an axial spatial resolution of 4.5 mm at 1 cm and 5.0 mm at 10 cm from the transverse FOV, a maximum sensitivity of 9–10 kcps/MBq at the centre of the FOV, and an axial FOV of 21.8 cm. All acquisitions were carried out in three-dimensional mode consisting of an emission scan of two minutes per bed position.

### 18F-FDG PET/MRI Protocol

PET/MRI were performed on Siemens Biograph mMR (Siemens Healthcare, Germany), which consisted of a 3-T MR scanner and simultaneous PET system without time of flight. Sequences were acquired from the skull vertex to mid thighs in a craniocaudal direction in three-dimensional mode with two and half minutes per bed position. PET acquisitions were assessed on an axial spatial resolution of 4.2 mm at 1 cm and 5.0 mm at 10 cm from the transverse FOV, a maximum sensitivity of 13.8 kcps/MBq at the centre of the FOV, and an axial FOV of 25.8 cm. Initial pre-treatment MRI sequences that were acquired including the following: axial 2-point Dixon 3-dimensional volumetric T1-weighted sequence before and after intravenous administration (0.1–0.2 mmol/kg body weight) of gadolinium-diethylenetriamine-pentaacetic acid contrast and coronal T2-weighted HASTE. Subsequent MRI imaging for the interim and end of treatment studies involved non-contrast sequences that involved axial 2-point Dixon 3-dimensional volumetric T1-weighted sequence, axial T2-weighted HASTE and coronal STIR. Given the safety of gadolinium-based contrast agents related to concentration-dependent deposition of gadolinium, a decision to omit gadolinium contrast for interim and end-of-treatment studies was made in view of the research nature of this study. Informed patient consent was obtained for all patients prior to each imaging interval.

The comparative total time of acquisition for each modality based on a calculated torso acquisition required 7 bed positions on the PET/CT, while the PET/MR required 5 bed positions to cover the same distance due to the smaller overlap and the larger axial field of view. Given this, the PET/CT acquisition time was 14 min (based on 2-min bed positions) and the PET/MR acquisition time was 12.5 min (based on 2.5-min bed positions). CT and MR protocols were incorporated into these acquisition times based on the relevant sequences obtained.

The design of the molecular imaging department at our institution incorporates both the PET/MR and the PET/CT rooms adjacent to each other and both are well shielded for 511 keV photons. Patients undertook a quiet resting state prior to and in between imaging modalities in dedicated individual shielded uptake rooms, which preserved individual patient separation and minimised exposure to departmental staff despite the high clinical through-put. The increased overall patient-staff interaction for setting up the patient on the PET/MR after the PET/CT was considered insignificant to overall exposure. The overall radiation exposure to technicians and nursing staff was monitored using both optically stimulated luminescence badges read monthly and electronic personal dosimeters with immediate readout and no higher-than-normal doses to any staff members were identified. Patients were encouraged to void prior to both imaging modalities and to mobilise independently off the imaging gantry where clinically practical.

### PET Image reconstruction

For both modalities, the PET data was corrected for randoms, dead time, scatter and attenuation. Reconstruction was performed using an iterative ordered-subset-expectation–maximization (OSEM) iterative algorithm with three iterations and 21 subsets. For the PET/CT, the low-dose CT data allowed PET attenuation correction using bilinear transformation with a 200 × 200 matrix. For the PET/MRI, attenuation correction was performed using attenuation maps generated from Dixon sequences using four distinct tissue categories (background, lungs, fat, soft tissue) obtained from Dixon fat and water weighted images with a 344 × 344 matrix. The PET/CT incorporated time-of-flight feature in PET reconstructions; however, this was not available on the PET/MR.

### Image reporting

Images were reported by senior dual-trained radiologists/nuclear medicine physicians (each with greater than 10 years’ experience). In total, six nuclear medicine physicians were involved in reporting. They were aware of the clinical history and prior imaging. To minimise bias, reporting physicians were blinded to same day image acquisitions with one reporting the PET/CT and another reporting the PET/MR.

### Diagnostic performance analysis

Diagnostic performance was assessed using three methods. Firstly, rates of detection of disease sites at staging, interim and end of treatment assessment were documented using PET/CT as the reference standard. Secondly, interim and end of treatment Deauville scores were compared between PET/CT and PET/MRI. Thirdly, concordance in metabolic activity (SUV max/SUV mean) in physiological and pathological tissue was assessed between PET/CT and PET/MRI at staging, interim and end of treatment assessments.

### Disease site detection

Differences between the PET/CT and PET/MRI were reviewed using a similar protocol to Verhagen et al. [17] and Latifoltojar et al. [22]. The disease status for 20 nodal and 10 extra nodal sites (a maximum of 30 sites per assessment) was assessed at staging, interim and end of treatment to document the presence or absence of involvement for each modality. The 20 nodal disease sites were Waldeyer’s ring, right cervical, left cervical, right supraclavicular, left supraclavicular, right axillary, left axillary, mediastinal, right hilar, left hilar, retrocrural, splenic hilar, liver hilar, spleen, mesenteric, retroperitoneal, right iliac, left iliac, right inguinal and left inguinal. The 10 extra-nodal disease sites were lung, pleura, peritoneum/omentum, gastrointestinal tract, liver, pancreas, kidney, breast, muscle, subcutaneous/soft tissue and bone. Nodal and extra-nodal involvement was defined as a focal area of FDG uptake with a SUV maximum (SUV max) above background mediastinal blood pool SUVmax or greater than the surrounding background in a location incompatible with physiological activity. Staging was performed using the modified Ann Arbor staging system [1].

### Response assessment

Residual metabolic activity at interim and end of treatment assessment was reported using the Deauville score. Complete metabolic remission was defined as a score of 1–3, as per international lymphoma guidelines [1].

### Physiological and pathological metabolic activity

Comparison of metabolic activity using SUVs in physiological and pathological tissue was performed using dedicated software for hybrid imaging (syngo.via; Siemens Medical Solutions, Germany). At each imaging time point, the dose of 18F-FDG and time between injection and PET/CT and PET/MR was recorded. For physiological tissue, maximum and mean SUVs were recorded for mediastinal blood pool activity in the descending thoracic aorta and background liver activity. These physiological SUV measures were generated using an automated software function.

The most avid pathological nodal and extra-nodal lesions were identified on staging imaging using the PET/CT as the reference standard. Where multiple disease sites were present in the same body region, only the largest from that body region was included for analysis. Up to four lesions were recorded for each subject. SUV values were determined by drawing a 3D-isocontour based on fused PET/CT images using a threshold of 41% SUV Max. The same disease site was then identified on PET/MRI with SUV parameters recorded using the same 3D-isocontour technique. The same process was undertaken for interim and EOT studies using the pathological sites identified in the initial staging study. Nodal metabolic activity was recorded in nodes even if the nodes had physically reduced in size to allow for recording of metabolic concordance. If, however, the initial disease site on the initial study was not visualised, then this was recorded as a resolved lesion.

### Radiation exposure

#### FDG PET effective dose

The average administered activity for this study, together with the biokinetic data for 18F FDG provided in ICRP 128 [23], were input into IDAC 2.1 internal dosimetry software [24] to calculate the contribution to effective dose (ED) from the PET component of the scan.

#### CT effective dose

CT-Expo v2.5 [25] was used for CT dose estimation. The typical scan parameters for a 60 kg female and 70 kg male scanned on the department’s Biograph 128 mCT scanner were calculated to match the female and male phantom sizes used by CT Expo. Organ doses were extracted from CT Expo for both the female and male exposures, and the effective dose was calculated using the ICRP 103 [26] methodology.

#### Risk estimates

The lifetime attributable risk of incidence for all cancers, published in Table 12D-1 of the BEIR VII Phase 2 report from the National Research Council [27], was used to estimate the risks associated with the effective dose from the PET and CT exposures. This was estimated for both sexes and the age range included in this study.

#### Statistical analysis

Detection rates, sensitivity and specificity with 95% confidence intervals (CI) were calculated for nodal and extra-nodal sites. The Kappa statistic was used to test agreement between modalities for disease site detection rates and Deauville scores. Agreement was classified as: none κ < 0, slight κ 0–0.2; fair κ 0.21–0.4; moderate κ 0.41–0.6; good κ 0.61–0.8; excellent κ 0.81– 1 [28]. Correlation between SUV parameters was assessed using intraclass correlations (ICC) estimates and their 95% CI, which were calculated based on a mean-rating (k = 2), consistency, 2-way mixed-effects model. Based on the 95% CI of the ICC estimate, values less than 0.5, 0.5–0.75, 0.75–0.9 and > 0.90 were indicative of poor, moderate, good, and excellent reliability, respectively [29]. The Wilcoxon signed rank test was used to test for any significant difference between the metabolic parameters in pathological sites at staging between the two modalities. The level of significance was set at *p* < 0.05. Statistical analysis was performed using R Core Team (2020) (R: A language and environment for statistical computing. R Foundation for Statistical Computing, Vienna, Austria. URL https://www.R-project.org/).

## Results

**Fig. 1:**
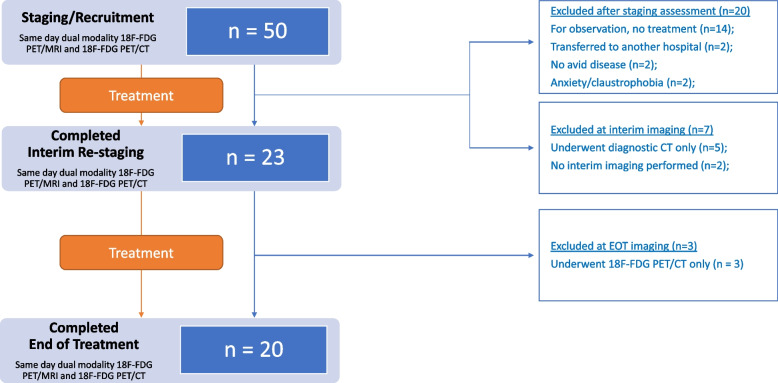
Study flow chart

A total of 50 patients were recruited. Baseline patient characteristics are shown in Table 1. The median age was 56 (range 21–80). The commonest lymphoma subtype was Hodgkin lymphoma (*n* = 14, 28%). The subtypes represented a range on indolent and aggressive lymphomas. A flow chart of patient recruitment is presented in Fig. 1. In total, 90 same-day dual modalities studies were assessed, which included the 50 staging studies and the 20 patients who completed interim and end of treatment imaging. Three patients completed interim but not end of treatment imaging. The commonest reason for patients not undergoing interim or end of treatment imaging was observation as a management strategy for indolent lymphoma. The median time between FDG administration and commencement of PET/CT was 60 min (interquartile range, 58—70 min). The median time between FDG administration and commencement of PET/MRI was 90 min (interquartile range, 80—99 min). Mean 18-FDG dose was 298.97 ± 58.09 MBq.

**Table 1:** Baseline patient characteristics

**Total patients, n**	50
**Median age, years (range)**	56 (21–80)
**Sex (Females: Males)**	22:28
**Diagnosis, n (%)**	
Hodgkin lymphoma (HL)	14 (28%)
Follicular lymphoma (FL)	13 (26%)
Diffuse large B-cell lymphoma (DLBCL)	7 (14%)
Mantle Cell lymphoma (MCL)	4 (8%)
Low-grade B-cell lymphoma unclassifiable	4 (8%)
Breast Implant-Associated Anaplastic Large Cell Lymphoma (BIA-ALCL)	2 (4%)
Burkitt lymphoma	1 (2%)
Post-transplant lymphoproliferative disorder (PTLD)	2 (4%)
Others^a^	3 (6%)
**Ann Arbor Stage**	
Stage I	11 (22%)
Stage II	6 (12%)
Stage III	8 (16%)
Stage IV	24 (48%)
Other^b^	1 (2%)

^a^Others = One case each of monoclonal B-cell lymphocytosis (MBL) with a chronic lymphocytic leukemia (CLL) immunophenotype, small lymphocytic lymphoma, orbital mucosa-associated lymphoid tissue (MALT)

^b^A single case of suspected B-cell lymphoma which was subsequently confirmed as MBL with CLL immunophenotype

### Staging assessment

At staging, PET/CT detected 352 positive disease sites (nodal *n* = 321, extra-nodal *n* = 31) out of a potential 1500 sites across 50 patients. PET/MRI detected 339 out of 352 true positive sites (nodal *n* = 309, extranodal *n* = 30), 1148 true negative sites and 13 false negatives sites (Table 2). There was excellent intermodality agreement in the identification of disease sites between PET/MRI and PET/CT (κ = 0.976, *P* < 0.001). The extra-nodal site not detected by PET/MRI was a small pulmonary nodule (< 10 mm). The remaining 12 false-negative sites not identified on PET/MR occurred in seven patients with small-volume (< 1 cm) axillary, cervical, mediastinal and hilar nodes. This resulted in a sensitivity of 96% (95% CI, 94% to 98%) and specificity of 100.00% (95% CI, 99% to 100%), κ = 0.976 (*P* < 0.001) (Table 3). There were no discrepancies between PET/MRI and PET/CT in the modified Ann Arbor staging score with perfect intermodality agreement (κ = 1, *P* = 0). Baseline staging results were: stage I *n* = 11, stage II *n* = 6, stage III *n* = 8, stage IV *n* = 24. One patient was not staged as they had a subsequent diagnosis of a monoclonal B lymphocytosis with a chronic lymphocytic leukemia (CLL) immunophenotype.

**Table 2:** Sites of disease detection for PET/MR vs PET/CT at staging, interim and end of treatment assessments

**Error**	**Staging**		**Interim**		**EOT**	
	**PET/CT (n)**	**PET/MRI (n)**	**PET/CT (n)**	**PET/MRI(n)**	**PET/CT (n)**	**PET/MRI(n)**
TP	352	339	27	19	13	13
FP	NA	0	0	0	0	0
TN	1148	1148	573	573	587	587
FN	NA	13	0	8	0	0
Total sites	1500	1500	600	600	600	600

*TP* True-positive, *FP* False-positive, *TN* True-negative, *FN* False-negative, *NA* Not applicable

**Table 3:** Performance of disease detection for PET/MR vs PET/CT at staging, interim and end of treatment assessments

**Staging**	
Sensitivity	96% (95% CI, 94% to 98%)
Specificity	100.00% (95% CI, 99% to 100%)
Kappa	0.976 (*p* < 0.001)
**Interim**	
Sensitivity	70% (95% CI, 50% to 86%)
Specificity	100% (95% CI, 99% to 100%)
Kappa	0.819 (*p* < 0.001)
**End of treatment**	
Sensitivity	100% (95% CI, 75% to 100%)
Specificity	100% (95% CI, 99% to 100%)
Kappa	1.0 (*p* < 0.001)

### Interim disease site identification and response assessment

**Fig. 2:**
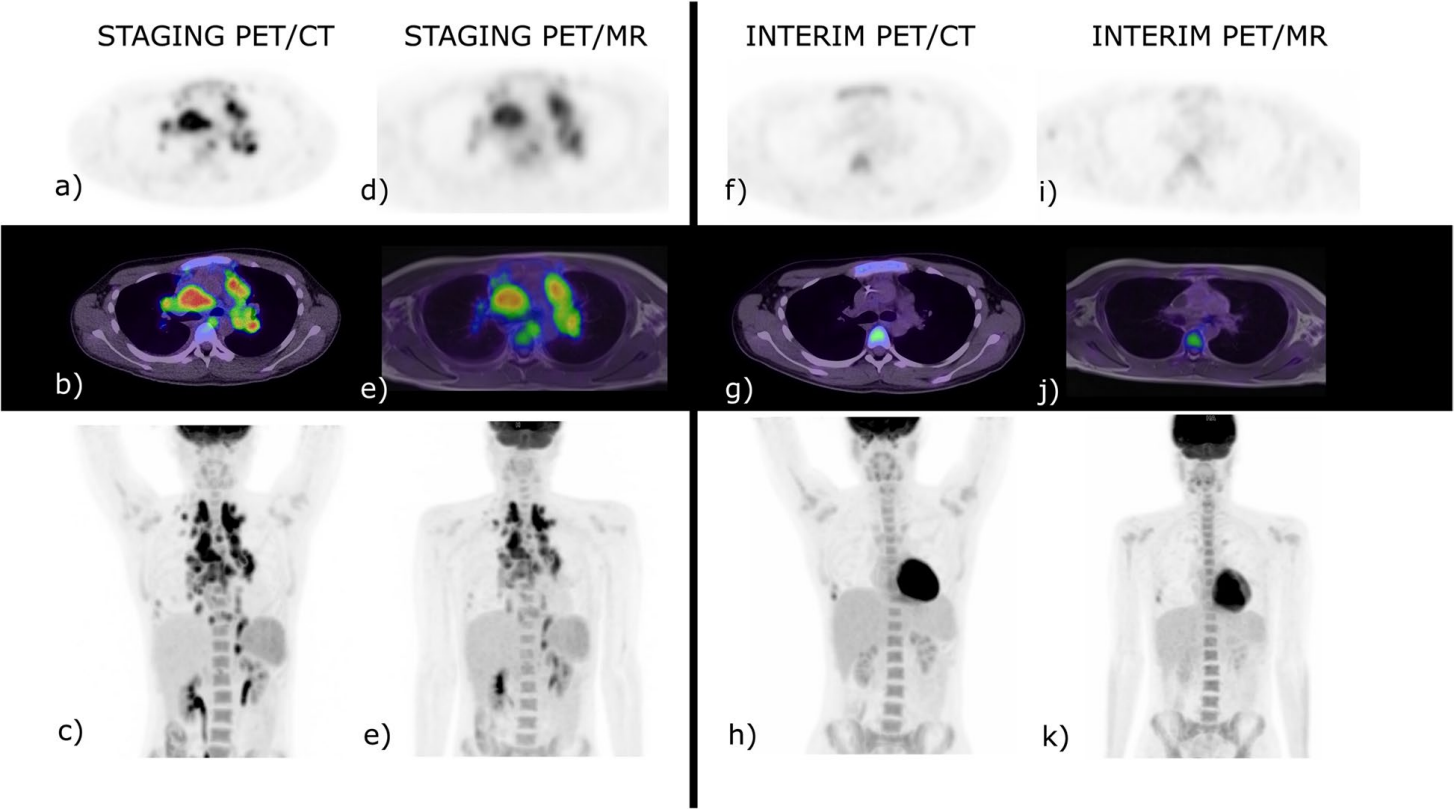
A 20-year-old male with biopsy proven classical Hodgkin lymphoma, stage IVB. Baseline PET/ CT (**a**-**c**) and baseline PET/MR (**d**-**e**). At interim re-staging PET/CT (**f**–**h**) and PET/MR (**i**-**k**) there was a concordant complete metabolic response, Deauville 2. The end of treatment study is not shown as there remained complete metabolic response. Incidental right-sided rib fractures persisted between staging and interim studies. SUV scale set between 0—8

Twenty patients were included in the total response assessment after completing interim and end of treatment assessment. At interim assessment, PET/CT identified 27 metabolically active sites (nodal *n* = 24, extra-nodal *n* = 3) out of a potential 600 sites in 13 patients. PET/MRI identified 19 of the 27 metabolic active sites (nodal *n* = 17; extranodal *n* = 2). This resulted in a sensitivity of 70% (95% CI, 50% to 86%) and specificity of 100% (95% CI, 99% to 100%), κ = 0.819, (*P* < 0.001) (Table 3). An example of concordance between modalities for a patient with complete metabolic response is demonstrated in Fig. 2.

**Fig. 3:**
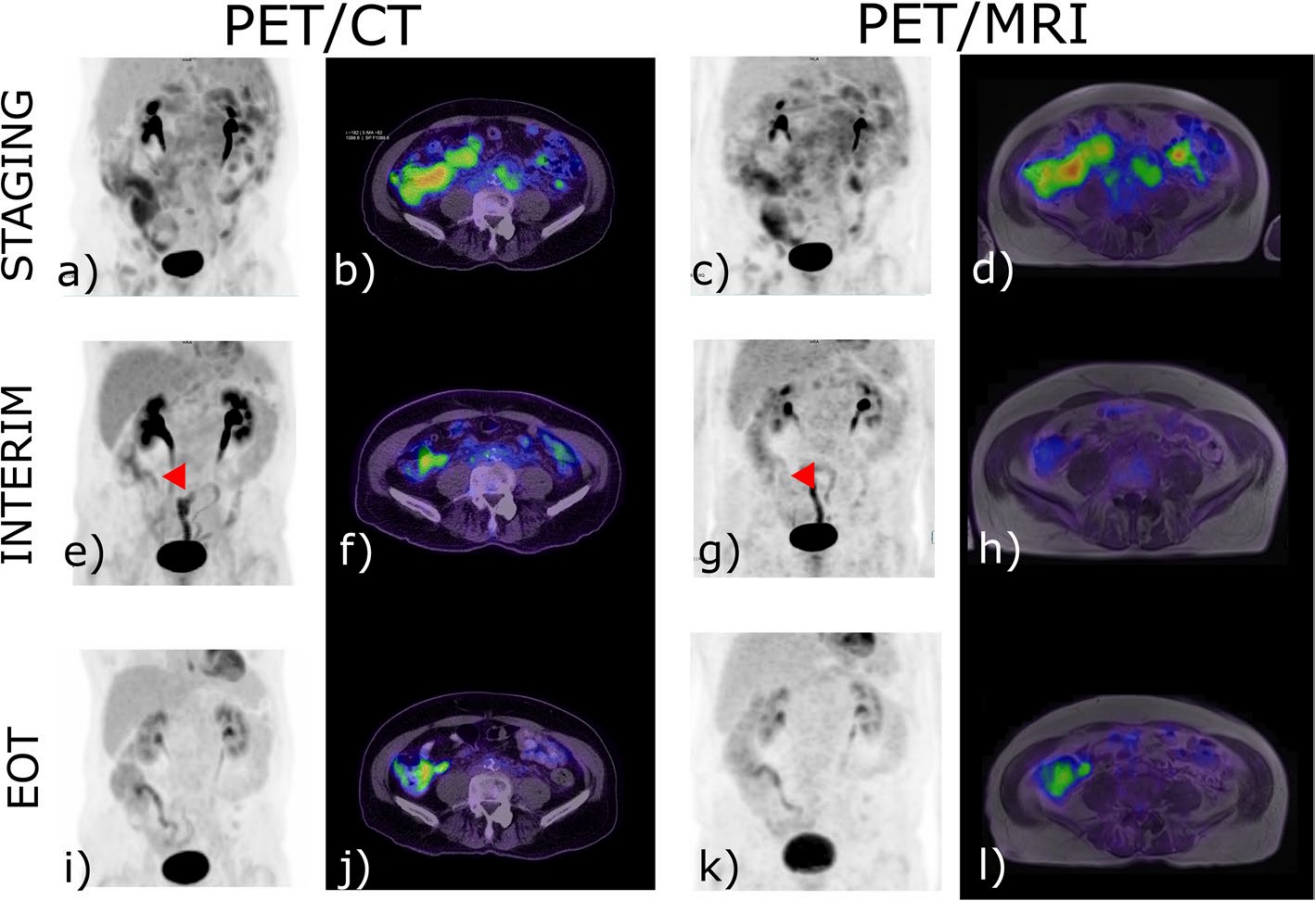
A 68-year-old male with biopsy proven Mantle cell lymphoma of the terminal ileum, stage IVA. At Baseline PET/ CT (**a**,**b**) and PET/MR (**c**,**d**) there was concordant avidity above that of background hepatic activity within the terminal ileum. At interim re-staging there remained uptake within the terminal ileum identified on the PET/CT (**e**,**f**) but not on the PET/MR (**g**,**h**) – red arrowhead. At end of treatment (EOT), there remained concordant persistent uptake in the terminal ileum. SUV scale set between 0—8

Eight sites were identified by PET/CT and not PET/MR in two patients, each with mantle cell lymphoma. In one patient, this related to differences in gastrointestinal tract FDG uptake between modalities in the setting of known gastrointestinal involvement. There was no underlying structural correlate identified on either cross-sectional modality (see Fig. 3). The second patient had small volume (< 1 cm) FDG-avid pulmonary hilar nodes identified on the PET/CT, which in this case was performed after the PET/MRI.

PET/MR demonstrated good intermodality agreement with 18 of 20 interim Deauville scores concordant (Deauville 2: *n* = 11, Deauville 3: *n* = 7), κ = 0.808 (*P* < 0.001) (Tables 4). The discordance in scores related to the two patients with mantle cell lymphoma where the SUV max of disease was lower on PET/MR than PET/CT for both patients (Deauville score 2 vs 4, respectively).

**Table 4:** Staging and response assessment results of PET/MR compared to PET/CT (reference standard)

**Staging (*n* = 50)**	PET/MR
Concordant	50
Upstaged	0
Downstaged	0
Intermodality agreement (kappa)	1.0 (*P* = 0)
**Interim (*n* = 20)**	
Concordant	18
Higher Deauville score	0
Lower Deauville score	2
Intermodality agreement (kappa)	0.808 (*P* < 0.001)
**End of treatment (*n* = 20)**	
Concordant	20
Higher Deauville score	0
Lower Deauville score	0
Intermodality agreement (kappa)	1.0 (*P* = 0)

### End of treatment disease site identification and response assessment

Twenty patients underwent end of treatment imaging. PET/CT identified 13 metabolically active sites out of 600 potential sites. PET/MRI identified all 13 sites that resulted in excellent intermodality agreement with a sensitivity of 100% (95% CI, 75% to 100%) and specificity of 100% (95% CI, 99% to 100%), κ = 1.0 (*P* < 0.001) (Table 3). Consequently, the Deauville score assessment was identical for both modalities: Deauville 2 *n* = 15, Deauville 3 *n* = 4, Deauville 4 *n* = 1 (κ = 1.0, *P* = 0). Of the 20 patients, 19 demonstrated complete metabolic response (Deauville score 1–3).

### Assessment of metabolic activity at physiological and pathological sites

Metabolic activity in 107 FDG-avid pathological nodal and extra-nodal disease sites was identified on the staging PET/CT studies with all sites detected on PET/MRI. There was excellent concordance in SUV max and mean between modalities in pathological uptake at staging (ICC 0.97 for both SUV max and SUV mean) with no significant mean difference (SUV max *P* = 0.30, SUV mean *P* = 0.33, Wilcoxon signed rank test). For physiological uptake, there was good concordance but the mean differences in SUV max values were consistently lower in PET/MR than PET/CT across all timepoints (Tables 5 and 6).

**Table 5:** Correlation between PET/MR versus PET/CT metabolic uptake values in physiological and pathological tissue across timepoints

	**Intraclass correlation coefficient (ICC)**	
	**Physiological Uptake**	**Pathological Uptake**
**Staging**		
SUVMax	0.85 (95% CI 0.77 – 0.90)	0.97 (95% CI 0.96 – 0.98)
SUVMean	0.80 (95% CI 0.70—0.86)	0.97 (95% CI 0.96—0.98)
**Interim**		
SUVMax	0.82 (95% CI 0.67 – 0.90)	0.91 (95% CI 0.82 – 0.95)
SUVMean	0.78 (95% CI 0.60–0.88)	0.94 (95% CI 0.89 – 0.97)
**EOT**		
SUVMax	0.83 (95% CI 0.68 – 0.91)	0.84 (95% CI 0.69 – 0.92)
SUVMean	0.85 (95% CI 0.71 – 0.92)	0.82 (95% CI 0.64 – 0.91)

**Table 6:** Absolute mean differences in metabolic uptake for PET/MR relative to PET/CT across imaging timepoints. Positive values indicate higher SUV in PET/MR. Negative values indicate higher SUV in PET/CT

	**Mean difference in metabolic uptake at PET/MR relative to PET/CT**			
	**Physiological uptake**		**Pathological uptake**	
	**SUVMax**	**SUVMean**	**SUVMax**	**SUVMean**
**Staging**	-0.23	-0.29	+ 0.19	+ 0.07
**Interim**	-0.27	-0.31	-0.22	-0.14
**EOT**	-0.37	-0.34	-0.32	-0.21

### Radiation dose

Estimated patient effective dose for the PET/CT incorporating CT-attenuation correction and PET resulted in an average calculated ED of 10.5 mSv per patient per scan. Average calculated ED for the PET/MR was 4.8 mV per patient per scan resulting in a difference of 5.7 mSv dose saving between the two modalities (54% dose saving). If an additional diagnostic CT of the neck, chest, abdomen and pelvis was performed with the PET/CT, the calculated average total ED would be 27 mSv. Based on the same aforementioned PET/MR dose, there would be a 22.2 mSv dose saving between the two modalities (82% dose saving) for each patient based on this scenario. Based on the calculated ED values, estimated age related risks of cancer incidence after an initial exposure to low-level ionizing radiation based on the Health Risks from Exposure to Low Levels of Ionizing Radiation BEIR VII Phase 2 [27] are presented in Table 7.

**Table 7:** Age related risks of cancer incidence from calculated radiation dose exposures from this study based on the Health Risks from Exposure to Low Levels of Ionizing Radiation BEIR VII Phase 2 [27]

Age	20	30	40	50	60	70	80
**PET/MR (calculated ED 4.8 mSv)**							
Male	1 in 2100	1 in 3000	1 in 3200	1 in 3500	1 in 4200	1 in 6000	1 in 12,000
Female	1 in 1300	1 in 1900	1 in 2300	1 in 2800	1 in 3500	1 in 5000	1 in 9600
**PET/CT (calculated ED 10.5 mSv)**							
Male	1 in 980	1 in 1400	1 in 1500	1 in 1600	1 in 1900	1 in 2800	1 in 5500
Female	1 in 580	1 in 900	1 in 1100	1 in 1300	1 in 1600	1 in 2300	1 in 4400
**PET/CT with additional diagnostic**^a ^**CT (calculated ED 27 mSv)**							
Male	1 in 380	1 in 540	1 in 570	1 in 630	1 in 760	1 in 1100	1 in 2100
Female	1 in 220	1 in 350	1 in 420	1 in 500	1 in 630	1 in 900	1 in 1700

^a^Diagnostic CT implies CT of the neck, chest, abdomen and pelvis

## Discussion

We prospectively compared the diagnostic performance at staging and response assessment of same day PET/MRI to PET/CT in lymphoma. All newly diagnosed patients with lymphoma were invited to participate in this study. Therefore, we captured a range of indolent and aggressive lymphomas. At staging, there was excellent agreement in the identification of nodal and extranodal sites between PET/MRI and PET/CT and complete concordance in Ann Arbour staging. There was good interim and excellent end of treatment intermodality agreement for disease sites and Deauville scores. This represents one of the first assessments of Deauville scores in an adult cohort of lymphoma patients across indolent and aggressive lymphomas. There was good–excellent concordance in SUV max and mean between modalities across timepoints. This data demonstrates the comparable accuracy in the identification of disease sites across multiple timepoints and objective response assessment with Deauville scores with PET/MRI compared to PET/CT. Importantly, we quantified the minimum radiation dose reduction with PET/MRI compared to PET/CT. With the benefit of reduced exposure to radiation, our data demonstrates that PET/MRI could be a practical alternative to PET/CT in the routine management of lymphoma.

Our data is consistent with prior reports with a high concordance in intermodality identification of sites of disease at staging using PET/MRI in lymphoma (k = 0.976) [13–15, 17, 18, 20, 30]. The false negatives on PET/MRI in small volume (< 1 cm) nodal tissue demonstrated as avid on the reference PET/CT could represent reactive tissue identified by the methodology as the same-day PET produced a dual-time point effect [31–33]. Importantly, these PET/MRI false negatives did not change staging. Correct staging is vital as it determines prognosis and guides treatment. Patients with limited stage disease (stage I or II) may be offered radiotherapy with curative intent. Conversely, it would be futile to offer curative intent treatment to a patient incorrectly staged with limited stage disease. There was good correlation of disease site identification at interim assessment and excellent correlation at end of treatment assessment.

Similarly, good (k = 0.819) and excellent (k = 1.0) agreement in PET/MRI compared to PET/CT Deauville scores was found with interim and end of treatment assessments, respectively. Interim Deauville scores were concordant for 18 of 20 cases and discordant for two (both Deauville scores 4 on PET/CT and 2 on PET/MR). For these discrepant cases, as the sites were not biopsied, it is not possible to determine if they represented lymphoma. One case involved gastrointestinal tract involvement where the differences between modalities may reflect the challenges of gut assessment with FDG-PET related to a combination of peristaltic activity, gastrointestinal lymphoid tissue and excreted radiotracer within the bowel lumen [34]. Interestingly, both these patients had mantle cell lymphoma, where the use of interim Deauville scores does not govern response-adapted therapy. A key benefit of PET/MRI relative to PET/CT relates to the superior soft tissue contrast of MRI sequences. Conversely, a known limitation relates to pulmonary nodules, with superior air-to-tissue contrast on CT. In this study a small pulmonary nodule (< 10 mm) identified on PET/CT was not identified on PET/MRI at staging. The inability of PET/MRI to demonstrate an avid lung lesion in a patient reflects the limitation in both MRI and non-time of flight PET sequences in the detection of small pulmonary lesions below 10 mm, which has been previously demonstrated [35, 36]. The limitation of pulmonary assessment on PET/MRI relates to a combination of factors including low proton density in aerated lungs, decay of signal caused by susceptibility artifacts at air–tissue boundaries, and motion artifacts caused by breathing and cardiac pulsation. This study did not encounter hepatic lymphoma deposits however superior MRI soft tissue contrast would benefit hepatic lesion assessment although would be dependent on MR sequence parameters. The end of treatment Deauville scores were concordant between modalities for each of these patients. Consequently, none of the patients would have been treated differently based on PET/MRI Deauville scores. These results are consistent with recent reports of concordant Deauville scores in paediatric [17] Hodgkin lymphoma (excellent k = 0.83) and adult Hodgkin [15] lymphoma (good k = 0.72) where discrepancies did not alter treatment decisions.

There was a high concordance in SUV max and mean between PET/MRI and PET/CT across timepoints. There was no significant difference in SUV max across pathological sites at staging between modalities although there was a relative mean increase in SUV units with PET/MRI relative to PET/CT at staging, which has been demonstrated previously [15]. Part of this may reflect the accumulation of FDG in malignant tissue over the second hour following radiotracer administration, a phenomenon which has been used to further delineate malignant lesions in PET imaging [31–33, 37]. Conversely, prior studies using dual-modality imaging in lymphoma have demonstrated lower PET/MRI SUVmax values using similar protocols to this study [14, 17, 20]. These factors may affect lesion conspicuity. Prior studies have observed underestimation of SUVs by PET/MRI not explained completely by differences in FDG injection timing that has been postulated to reflect differences in underlying attenuation-correction algorithms [16, 38, 39]. The concordance in metabolic parameters in physiological tissues was expected, with the relative mean reduction in SUV units between PET/MRI and PET/CT in keeping with physiological blood distribution and hepatic handling of glucose with time [21].

Radiation exposure from medical imaging may induce cancer. A study in patients with HL and DLBCL reported a cumulative radiation risk of 1 excess cancer per 100 patients from imaging performed during the first year after diagnosis and over 5 years of subsequent follow-up [40]. In our study, the lack of additional ionizing radiation from PET/MRI reduced the overall exposure with calculated radiation dose savings of 54% compared with the PET/CT with attenuation correction low-dose CT and 82% relative to a PET/CT performed with an additional concurrent diagnostic CT. This is of particular importance in younger patients with indolent lymphomas who would require indefinite surveillance imaging, with relatively increased lifetime risk of cancer incidence relative to older populations.

This study has several limitations. There was an unavoidable delay in performing PET/MRI with a single FDG injection. Variations in FDG metabolism could affect disease detection due to differences in metabolism in normal versus malignant tissue resulting in an improved contrast-to-noise ratio, particularly on the staging PET/MRI. However, in this study, there were no false positives at any time point. Alternatively, the prolonged uptake time could affect SUV parameter measurements due to decay of the isotope, which may explain the two discrepant Deauville scores. The distribution of histological subtypes limits formal subgroup analysis; however, the consistency of findings across indolent and aggressive lymphomas suggests the conclusions hold true. Furthermore, the distribution of histologies represents the spectrum of clinical practice. The retention rate from initial recruitment at staging to end of treatment assessment reflects a combination of therapeutic management (observation in some cases of indolent lymphoma), the loss of patients who relocated or patients who declined further PET/MRI due to claustrophobia. This study was performed in a single tertiary referral centre. Given the contemporary nature of PET/MRI, studies with multiple institutions with standardized imaging protocols would allow further assessment of inter-equipment variability and would benefit from a larger recruitment population.

## Conclusion

PET/CT represents the current standard in lymphoma due to availability and familiarity. For PET/MRI to be introduced into clinical practice, diagnostic performance is a crucial issue. Our data supports and extends prior work reporting comparable performance in the detection of sites of disease. Furthermore, we found comparable performance in the critical issue of Deauville scores across indolent and aggressive lymphomas. Future studies should aim to identify novel prognostic biomarkers from multiparametric MRI. This would guide therapeutic decisions at diagnosis and at interim and end of treatment response assessment timepoints. With the benefit of avoiding radiation exposure, PET/MRI appears an attractive alternative to PET/CT across indolent and aggressive lymphomas with comparable performance.

## Data Availability

The datasets used and analysed during the current study are available from the corresponding author on reasonable request.

## Abbreviations

CI: Confidence intervals
CT: Computed tomography
DLBCL: Diffuse large B-cell lymphoma
ED: Effective dose
FDG: Fluorodeoxyglucose
HL: Hodgkin lymphoma
ICC: Intraclass correlations
MRI: Magnetic resonance imaging
PET: Positron emission tomography
SUV: Standardised uptake values
